# On Criticality for a Generalized Couette Flow of a Branch-Chain Thermal Reactive Third-Grade Fluid with Reynold's Viscosity Model

**DOI:** 10.1155/2020/7915954

**Published:** 2020-11-10

**Authors:** S. O. Salawu, A. B. Disu, M. S. Dada

**Affiliations:** ^1^Department of Mathematics, Landmark University, Omu-Aran, Nigeria; ^2^Department of Mathematics, National Open University of Nigeria, Abuja, Nigeria; ^3^Department of Mathematics, University of Ilorin, Ilorin, Nigeria

## Abstract

This research considers the third-grade liquid flow and criticality branched-chain of a thermal reaction in a Couette generalized medium with a nonlinear viscosity model. A dimensionless transformation of the system momentum and heat equations are carried out. Compared with the diffusion coefficient, the flow is stimulated by initiation reaction rate, reaction branch-chain order, non-Newtonian term, thermal Grashof number, and pressure gradient. The reactive fluid is fully exothermic with consumption of the material, and the heat exchange in the system is greater than the exchange of heat with the ambient. A semianalytical collocation weighted residual scheme is employed for the branch-chain slice bifurcation, dimensionless energy, and momentum solutions. The results show that exponential decreases in the thermal fluid viscosity can help in controlling the boundless heat produced by the Frank-Kamenetskii term and initiation reaction rate. Therefore, the results will help in stimulating positive combustion processes.

## 1. Introduction

Limited industrial usage of Newtonian fluid has substantially encouraged the interest in non-Newtonian fluid and its applications in science, technology, and manufacturing processes such as polymer film production and fiberglass. The flow of liquids under the action pressure gradient in a device with the moving part where lubrication takes place described Couette generalized flow [1, 2]. Non-Newtonian fluid with viscoelastic characteristics can help in improving lubricants of industrial machines and technology devices' efficiency. As such, Nayak et al. [3] examined entropy optimization of the hydromagnetic non-Newtonian nanomaterial with a joint approach for heat transfer intensification and solar energy absorber. The problem was solved numerically, and the result shows that a rise in the Weissenberg number enhanced fluid velocity. Wang et al. [4] reported on the non-Newtonian reaction catalyst of the heterogeneous-homogeneous Oldroyd fluid with radiation and heat absorption. From the study, it was revealed that radiation and Biot number have a direct relation to the temperature distribution. With heat transfer, it is known that shear rate and friction between the fluid and moving devices can lead to significant heat production in the system that may affect the device and fluid properties [5–8]. Among the liquids that exhibit viscoelastic characteristics is the third-grade liquid. Third-grade fluid model is a non-Newtonian liquid model that predicts shear thickening or thinning properties over a definite boundary.

Due to its applications, Yilbas et al. [9] examined entropy production of the third-grade liquid with unvarying viscosity in an annular pipe using analytical approximate solutions for the entropy generation and other flow properties. It was reported that improved non-Newtonian material terms will assist in reducing the irreversibility process. Yurusoy et al. [10] improved on the work of Yilbas et al. [9] by considering Vogel model viscosity and solved the steady third-grade fluid flow in a concentric rigid cylinder by the perturbation method. It was noticed that heat-dependent viscosity supported increasing the velocity field. In a convective cooling channel, the irreversibility of the flow of the third-grade reactive liquid with dissipative viscous heating was carried out by Salawu and Adesanya et al. [11, 12]. The solutions to the equations were obtained using semianalytical techniques, and it was reported that the non-Newtonian term decreases the flow rate. Okoya [13] studied computationally the nonlinear viscosity of the third-grade reactive fluid with heat effect on axial annular flow. It was revealed that the reactive third-grade and Newtonian fluids are the same qualitatively in an annulus pipe except for the level of inward translocation of the peak temperature and axial momentum. Khan et al. [14] considered modeling and simulation of the micropolar ferrofluid for slip velocity of second order with saturated permeable media. It was found that the fluid material terms decrease the fluid velocity due to simulation in the liquid viscosity. However, in a reactive combustion heat transfer process without heat diffusion, exothermic reaction will reach the state of criticality no matter the starting temperature [15, 16].

Thermal criticality performs a significant role in processing and handling of the non-Newtonian fluid. It exists when the heat generation rate in a reactive flow system transcends heat dissipation to the environments [17, 18]. This occurrence is the basis for thermal ignition or runaway in a flow system, Frank-Kamenetskii [19]. The main objective of analyzing thermal criticality is to predict the unsafe or critical state of an exothermic reaction flow condition in a combustion process. Combustion is absolute and essential for the chemically reacting flow system with applications in pollution control, power generation, processing material industries, and so on, Balakrishnan et al. [20]. As a result of its importance, Salawu et al. [21] investigated the irreversibility and thermal criticality of Powell–Eyring reactive liquid flow with radiation and variable conductivity in permeable media. The authors revealed that thermal explosion can be avoided in a reactive system if heat source terms are minimized. Makinde and Maserumule [5] examined thermal ignition and the second law of Couette fluid flow with variable viscosity. Analytical solutions of the problem were provided using perturbation techniques along with the Hermite approximation method. The results obtained proved clearly that the Frank-Kamenetskii term is a strong internal heat generation term that leads to thermal criticality or ignition. In a porous-filled channel, analytical solution of viscous reactive thermal ignition in a slab was carried out by Makinde [22]. In the study, essential properties of heat distribution and criticality slice bifurcation were reported. Also, with series of solution technique, thermal explosion of exothermic reaction in a slab was examined by Makinde [23]. The study presented that energy reactive parameters can assist in controlling exothermic reaction combustion explosion.

This study is built based on Okoya [16] which is an extension of the work done by Varatharajan and Williams [24] in which branch-chain transition and criticality in a slab are considered. The indisputable results obtained in their studies and suggested further extension have motivated this investigation. This study presents coupling fluid flow and heat transfer to investigate the bifurcation slice for the thermal criticality of the third-grade reactive Couette fluid with nonlinear viscosity and heat distribution. Therefore, it is necessary to study the criticality of the branched-chain thermal-reactive diffusion problem in order to obtain competing effects of emerging parameters and enhance its utilization in different manufacturing industries. This study is essential in determining combustion system capability under different operating conditions in order to improve safety; it will also help in decreasing combustion product pollution. The considered problem is solved by weighted residual techniques coupled with the collocation scheme. Results from the method used are observed to quantitatively and qualitatively agree with the computed results.

## 2. Problem Formulation

**Figure 1:**
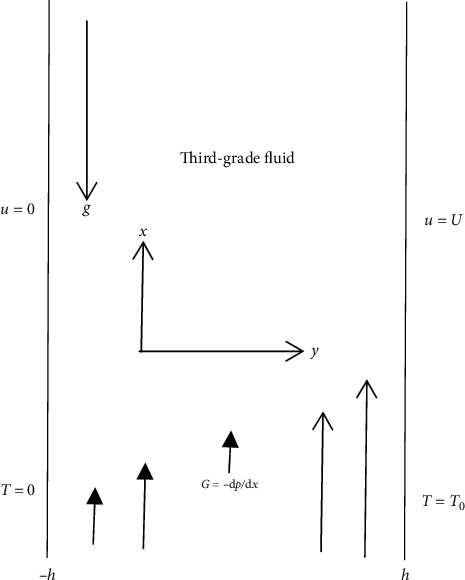
Flow schematic coordinate.

Consider the flow of the reactive mixture in a Couette generalized device (Figure 1) bounded with isothermal walls. The flow has a unidirectional velocity field with Reynold's viscosity under the influence of gravity, pre-exponential factor *m*, and *n* order of reaction branching. The isothermal flow configuration is positioned at *y* = [−*h*; *h*] with *y*-axis normal to the flow direction in the *x*-axis, and the non-Newtonian formulation is used to create the viscoelastic effects. The branched-chain parameter is described in the form of the reaction rate generalized law as

(1) $$\begin{array}{c} P=P_{0}{\left(\frac{Tk}{\nu \hbar }\right)}^{m}\exp\left(-\frac{E}{RT}\right). \end{array}$$

The consumption chemical reactant is taken to be very small with constant coefficients. The terms *P*_0_, *ℏ*, *k*, *E*, *m*, *n*, *ν*, *T*_0_, and *T* correspond to the branch-chain rate, Planck's number, Boltzmann constant, activation energy, pre-exponential constant, reaction branch order, frequency of vibration, initial heat, and fluid heat. The momentum single-fluid balance for the dynamic species is given as follows (Truesdell [25]):

(2) $$\begin{array}{c} \rho \frac{\text{d}v}{\text{d}t}=\rho F+divT. \end{array}$$

Given that the chemical reaction does not create flow momentum, the kinematic-related variables for the third-grade fluid that are thermodynamically appropriate in the stress tensor form according to Fosdick and Rajagopal [26] can be expressed as

(3) $$\begin{array}{c} T=\mu B_{1}+r_{1}B_{2}+r_{2}B_{1}^{2}+c_{3}\left(trB_{1}^{2}\right)B_{1}-pI, \end{array}$$

where *μ* is the dynamic viscosity, **I** is the unit tensor, *p* is the pressure, and *tr* is the trace matrix. The terms *r*_1_, *c*_3_, and *r*_2_ are the variable temperature of the material coefficients defined as follows:

(4) $$\begin{array}{c} \mu \geq 0,r_{1}\geq 0,c_{3}\geq 0,\left|r_{1}+r_{2}\right|\leq \sqrt{24\mu c_{3}}. \end{array}$$

The kinematic tensors **B**_**1**_ and **B**_**2**_ can be expressed as

(5) $$\begin{array}{c} B_{1}={\left(\nabla v\right)}^{\text{T}}+\left(\nabla v\right),B_{2}=\left(\frac{d}{dt}\right)B_{1}+{\left(\nabla v\right)}^{\text{T}}B_{1}+B_{1}\left(\nabla v\right). \end{array}$$

The term ∇ is an operator (gradient), index *T* denotes transpose, **v** is the vector velocity, and the derivative material term is d/d*t*. The flow ensues in an erect device with a spontaneous velocity *U* as the chemical reaction is being prompted to raise proliferation of chain carriers. The reactive fluid accelerates in the medium close to the wall, while the other wall remains static.

Following the assumptions stated above and [16], the nondimensional steady velocity and energy balance equations are written as

(6) $$\begin{array}{c} G+\text{exp}\left(-a\theta \right)\left(\frac{{\text{d}}^{2}w}{\text{d}y^{2}}\right)-a\text{exp}\left(-a\theta \right)\left(\frac{\text{d}\theta }{\text{d}y}\right)\left(\frac{\text{d}w}{\text{d}y}\right)+6\Lambda \frac{{\text{d}}^{2}w}{\text{d}y^{2}}{\left(\frac{\text{d}w}{\text{d}y}\right)}^{2}+Gr\theta =0, \end{array}$$

(7) $$\begin{array}{c} \frac{{\text{d}}^{2}\theta }{\text{d}y^{2}}+\lambda \theta^{n}{\left(1+\epsilon \theta \right)}^{m}\text{exp}\left(\frac{\theta }{1+\epsilon \theta }\right)+Q=0. \end{array}$$

Here, the parameters *w* and *θ* depict nondimensionless flow velocity and energy. The terms *Q*, *m*, *ϵ*, *n*, *λ*, *Gr*, *G*, *a*, and Λ individually represent the initiation rate, pre-exponential factor, activation energy (that is, the energy needed for a species reaction mixture to take place; thus, it determines the transition state of a chemical reaction), branch-chain order, Frank-Kamenetskii (that is, homogeneous mixture of thermal ignition species at walls' constant temperature. It determines the species reaction time to heat conducting time), heat Grashof number, pressure gradient, variable viscosity, and non-Newtonian. The suitable nondimensional boundary conditions are

(8) $$\begin{array}{c} w\left(-1\right)=0,\\ w\left(1\right)=1,\\ \theta \left(-1\right)=0,\\ \theta \left(1\right)=0. \end{array}$$

The heat-dependent dynamic viscosity is assumed to exponentially vary according to Salawu et al. [17, 27]:

(9) $$\begin{array}{c} \bar{\mu }=\mu_{0}\exp\left(-\alpha \left(T-T_{0}\right)\right). \end{array}$$

The succeeding quantities are utilized to get nondimensional formulated models (6) to (8):

(10) $$\begin{array}{c} y=\frac{\bar{y}}{h},\\ \theta =\frac{E\left(T-T_{0}\right)}{RT_{0}^{2}},\\ \mu =\frac{\bar{\mu }}{\mu_{0}},\\ \varepsilon =\frac{RT_{0}}{E},\\ \lambda =\frac{\alpha_{0}h^{2}P_{0}E}{RT_{0}^{2}D}{\left(\frac{\varepsilon T_{0}}{\alpha_{0}}\right)}^{n}{\left(\frac{kT_{0}}{\hbar \nu }\right)}^{m}\exp\left(\frac{1}{\varepsilon }\right),\\ U=\frac{\mu_{0}}{\rho h},\\ p=\frac{\bar{P}\rho h^{2}}{\mu_{0}^{2}},\\ G=-\frac{d\bar{P}}{dx},\\ w=\frac{\rho hu}{\mu_{0}},\\ \Lambda =\frac{\mu_{0}\beta_{3}}{\rho^{2}h^{4}},\\ Gr=\frac{RT_{0}^{2}g\beta \rho^{2}h^{3}}{\mu_{0}^{2}E},\\ a=\frac{RT_{0}^{2}\alpha }{E},\\ Q=\frac{P_{0}B\alpha_{0}}{DT_{0}\varepsilon },\\ x=\frac{\bar{x}}{h}. \end{array}$$

### 2.1. Limiting Cases

When *Gr* = *n* = *Q* = 0, equations (6) and (7) correspond to the case of the nonlinear viscosity flow model with heat transfer investigated by Okoya [28], while the corresponding model with *Gr* ≠ 0 was considered by Salawu and Fatunmbi [11, 29]. In the absence of momentum and reaction branch order, the exceptional instance of *ϵ* = *Q* = 0 corresponds to the Frank-Kamenetskii traditional model [19], while the case *ϵ* ≠ 0 and *m* ∈ {0.5, 0, −2} was described was described by Boddington et al. [30].

## 3. Method of the Solution

The solution technique for the criticality bifurcation slice, temperature, and momentum equations is performed using a semianalytical technique. In the scheme, it is taken that

(11) $$\begin{array}{c} u\left(y,d\right)=\phi_{0}\left(y\right)+\sum_{i=1}^{n}d_{i}\phi_{i}, \end{array}$$

as defined in [31, 32], and *ϕ*_*i*_(*y*) is an assigned function with the boundary conditions satisfied. The basis function *u*(*y*, *d*) is individually illustrated for the boundary conditions and their respective equations. Hence, for the arbitrary chosen values of *d*′*s*, the residual equation is obtained as

(12) $$\begin{array}{c} W\left(y,d\right)=K\left(u\left(y,d\right)\right)-r\left(y\right). \end{array}$$

For the function *ϕ*_*i*_ in successful approximation, the differential equations are satisfied by *u*(*y*, *d*). The main aim is to minimize *W*(*y*, *d*) errors to zero (say) in between the domain, i.e.,

(13) $$\begin{array}{c} \int_{Y}W\left(y,d\right)V_{i}dy=0,i=1,2,..,n. \end{array}$$

The number of weighted functions *V*_*i*_ must be translated to the number of unknown constants *d*_*i*_′*s* in *u*. The integration collocation scheme is adopted for the solution in which the weight function is presented in Dirac delta as *V*_*i*_(*y*) = *δ*(*y* − *y*_*i*_) such that *W*(*y*, *d*) = 0.

The method is employed on boundary conditions (8) and on dimensionless equations (6) and (7) to have the following residual equations:

(14) $$\begin{array}{c} w_{r}=G+\exp\left(-a\left\{y^{10}b_{10}+y^{9}b_{9}+y^{8}b_{8}+y^{7}b_{7}+y^{6}b_{6}+y^{5}b_{5}+y^{4}b_{4}+y^{3}b_{3}+y^{2}b_{2}+yb_{1}+b_{0}\right\}\right)\times \left(90y^{8}a_{10}+72y^{7}a_{9}+56y^{6}a_{8}+42y^{5}a_{7}+30y^{4}a_{6}+20y^{3}a_{5}+12y^{2}a_{4}+6ya_{3}+2a_{2}\right)-a\times \exp\left(-a\left\{y^{10}b_{10}+y^{9}b_{9}+y^{8}b_{8}+y^{7}b_{7}+y^{6}b_{6}+y^{5}b_{5}+y^{4}b_{4}+y^{3}b_{3}+y^{2}b_{2}+yb_{1}+b_{0}\right\}\right)\times \left(10y^{9}a_{10}+9y^{8}a_{9}+8y^{7}a_{8}+7y^{6}a_{7}+6y^{5}a_{6}+5y^{4}a_{5}+4y^{3}a_{4}+3y^{2}a_{3}+2ya_{2}\right)+\ldots \end{array}$$

(15) $$\begin{array}{c} \theta_{r}=90y^{8}b_{10}+72y^{7}b_{9}+56y^{6}b_{8}+42y^{5}b_{7}+30y^{4}b_{6}+20y^{3}b_{5}+12y^{2}b_{4}+6yb_{3}+2b_{2}+\lambda {\left(y^{10}b_{10}+y^{9}b_{9}+y^{8}b_{8}+y^{7}b_{7}+y^{6}b_{6}+y^{5}b_{5}+y^{4}b_{4}+y^{3}b_{3}+y^{2}b_{2}+yb_{1}+b_{0}\right)}^{n}\times {\left(\varepsilon \left(y^{10}b_{10}+y^{9}b_{9}+y^{8}b_{8}+y^{7}b_{7}+y^{6}b_{6}+y^{5}b_{5}+y^{4}b_{4}+y^{3}b_{3}+y^{2}b_{2}+yb_{1}+b_{0}\right)+1\right)}^{m}+\ldots \end{array}$$

Within the domain, the collocation techniques are applied on equations (14) and (15) which are then solved together with the boundary conditions to get the coefficients *a*_*i*_′*s* and *b*_*i*_′*s*. The solution algorithms are repeated for various values of parameters. Maple software is used to determine the constant coefficients and solve the equations completely.

## 4. Discussion of Results

**Figure 2:**
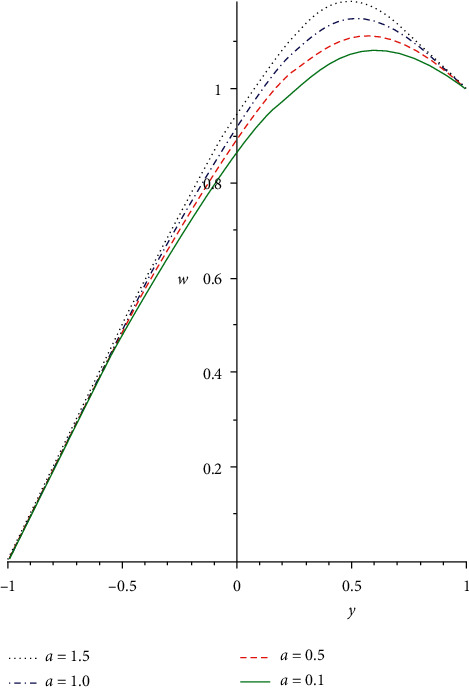
Velocity profile for rising values of *a*.

**Figure 3:**
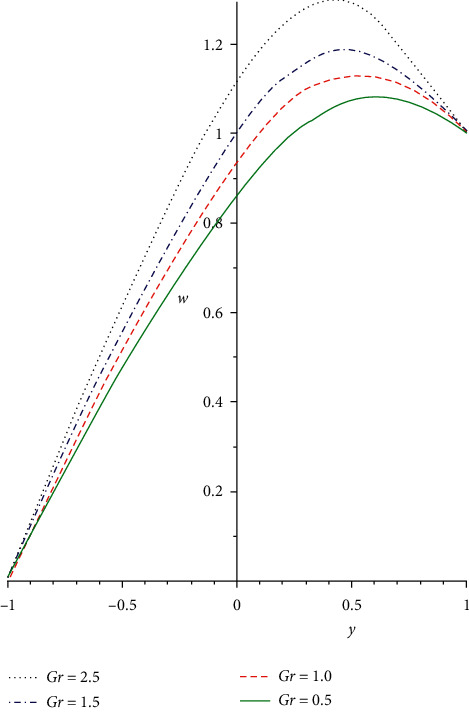
Velocity profile for increasing *Gr*.

**Figure 4:**
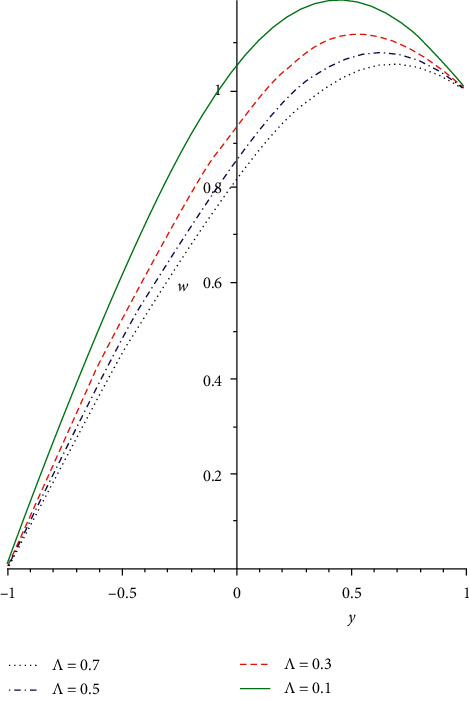
Flow rate field for various values of Λ.

**Figure 5:**
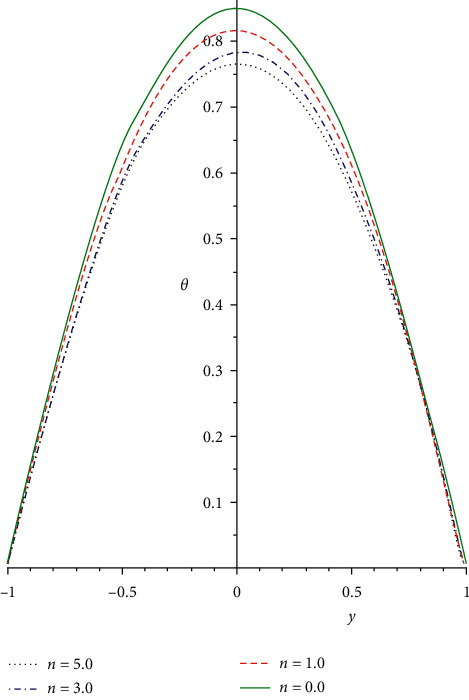
Heat profile for rising values of *n*.

**Figure 6:**
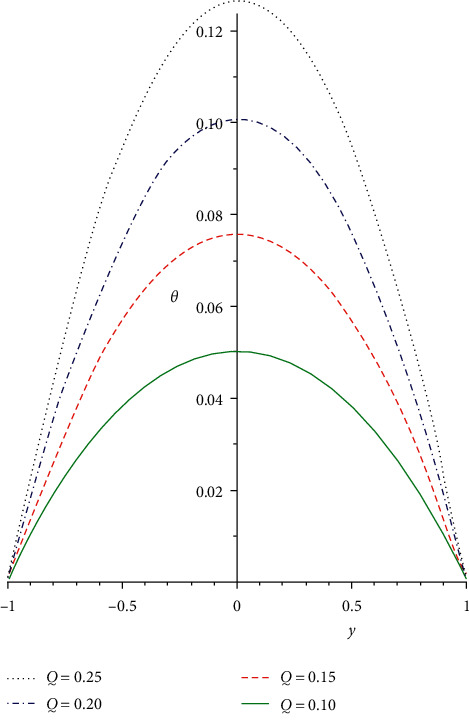
Heat field for rising values of *Q*.

**Figure 7:**
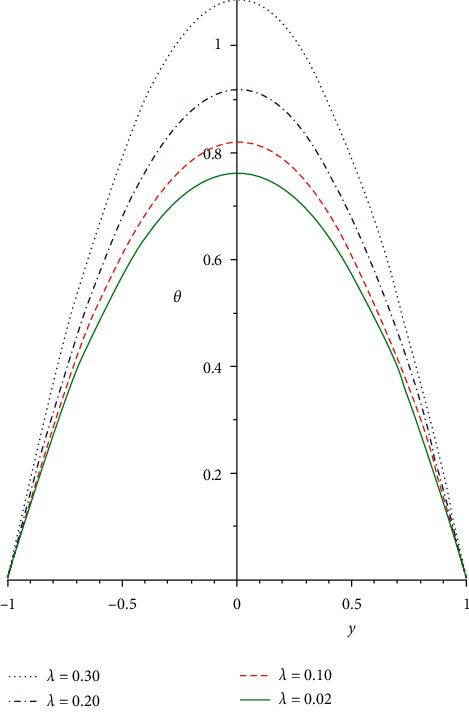
Heat profile for different values of *λ*.

**Figure 8:**
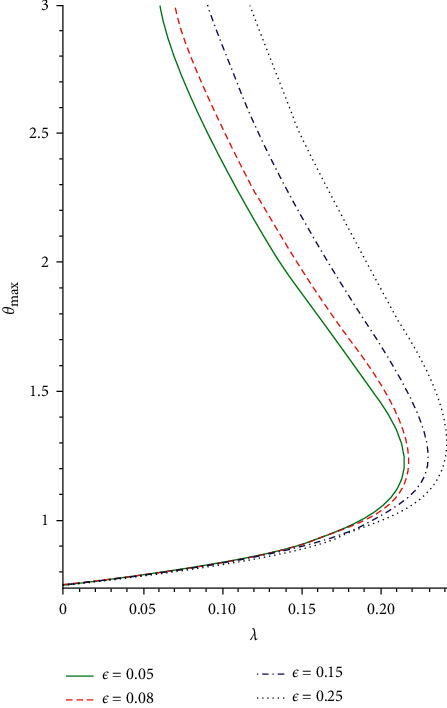
Criticality bifurcation for rising *ϵ*.

**Figure 9:**
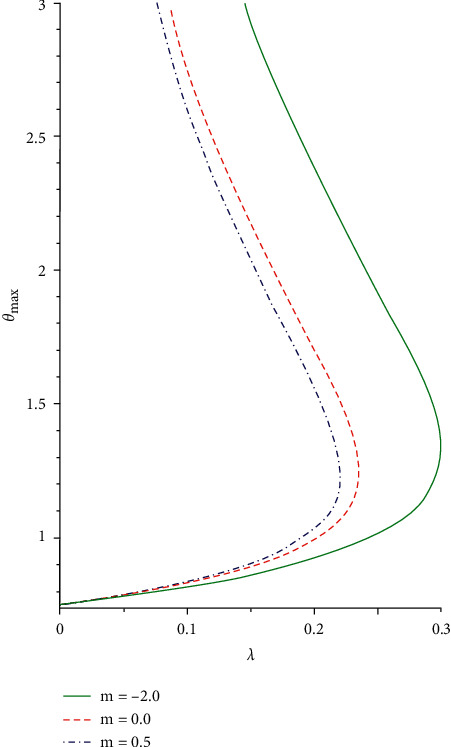
Criticality bifurcation for various *m*.

**Figure 10:**
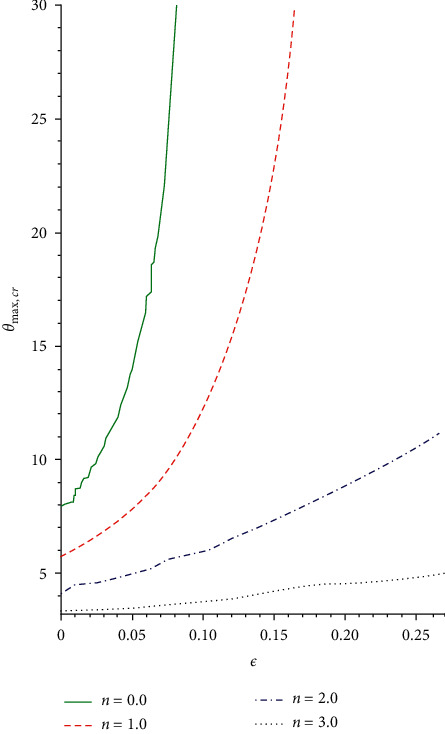
Plot of *θ*_max,*cr*_ against *ϵ* for rising *α*.

**Figure 11:**
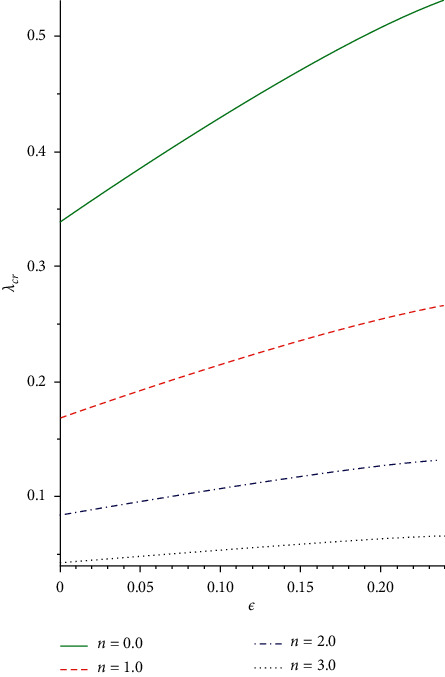
Plot of *δ*_*cr*_ against *ϵ* for rising *α*.

The solutions to the thermal criticality, velocity, and heat equations are obtained via the weighted residual collocation scheme. The computational default values are taken according to [33, 34]. The results obtained are presented as plots in Figures 2–11. The accuracy and consistency of the method are established by comparing it with the Fehlberg–Runge–Kutta scheme along with the shooting method (numerical method) as depicted in the table. In Table 1, the results for the weighted residual technique and numerical scheme are compared as presented. The semianalytical method used gives good solutions when relating with the numerical method results. The collocation WRM contracted well with the other solution approaches with the order of absolute error 10^−8^.

### 4.1. Velocity Profile for Parameter-Dependent Solutions

Figure 2 illustrates the impact of Reynold's viscosity term *a* on the velocity profile. As the values of the heat-dependent viscosity term increase, the fluid viscosity diminishes, and the fluid bonding force is discouraged. As such, the fluid molecular diffusion is boosted which correspondingly diminishes the flow resistance force. Hence, the non-Newtonian fluid particle collision is enhanced that leads to an increasing fluid velocity distribution. In Figure 3, the reaction of the non-Newtonian flow liquid to rising in the Grashof number *Gr* is demonstrated. The term *Gr* is the ratio of buoyancy to the viscous force exerting on a liquid which is equivalent to the Reynolds number. A rise in the thermal Grashof number enhances heat source terms which leads to a breakdown in the fluid bonding forces. The breaking down in the liquid bonding forces causes the fluid particles to move freely and thereby increases the flow rate.  Figure 4 shows the response of the third-grade liquid to variation in the non-Newtonian term Λ. Large shear rate occurs in a generalized Couette flow, which acts as a source term for the heat and velocity equations. In a third-grade liquid, the term Λ needs to be kept at small values to discourage flow opposition forces. Therefore, increasing the values of the term Λ decreases the flow velocity field due to a decrease in the source terms.

### 4.2. Temperature Field for Parameter-Dependent Solutions

Figures 5–7 depict the temperature distribution for the rising values of the terms *n*, *Q*, and *λ* for the function *θ*(*y*) plotted against *y*. The impacts of branch reaction order *n* on the temperature profile are displayed in Figure 5. The branch reaction order is a basis for thermal ignition; it entails steps of the reaction chain in which each step serves as a reagent for the next step. Therefore, rising the reaction branch order reduces the heat profile. This is as a result of heat diffusion from the device that causes general decreases in the system heat field. The influence of the rate of initiation *Q* and Frank-Kamenetskii term *λ* on the energy distribution is illustrated in Figures 6 and 7. Both parameters are high heat generation terms and must be carefully monitored to avert the blow up of the reactive solution. The temperature profile is enhanced at the fixed wall and consistently rises until it gets to the highest at the channel center. It then diminishes continuously close to the moving surface. Continuous decrease in the heat profiles is observed as the viscosity of the liquid reduces in a reactive exothermic third-grade liquid. From the viewpoint of technology design, total heat generation can be minimized if the viscosity of the liquid as a result of rises in heat can be exponentially reduced.

### 4.3. Criticality Branch-Chain and Ignition Solutions

The slice bifurcation thermal runaway plot for 0 < *ϵ* ≪ 1 and different pre-exponential factor *m* in the plane (*λ*, *θ*_max_) is demonstrated in Figures 8 and 9. The diagrams depict the qualitative difference in the reactive non-Newtonian liquid as the term *λ* increases. In fact, for 0 ≤ *ϵ* ≪ 1 and *m* = −2,0,0.5, critical value *λ*_*cr*_ occurs so that, for 0 < *λ* < *λ*_*cr*_, two solution branches are found. The lower solution branch is steady, while the upper branch solution diverges to infinity as *λ*⟶0. When *λ* > *λ*_*cr*_, there is no real solution but a traditional form demonstrating temperature criticality. A rise in the activation energy *ϵ* raises the system thermal explosion but declines the system heat steadiness as the chemical kinetics *m* is encouraged as described in Figures 8 and 9. The considerable variations in the maximum heat (*θ*_max,*cr*_) and ignition criticality (*λ*_*cr*_) for a third-grade reactive flow as the parameter *ϵ* rises are confirmed in Figures 10 and 11. An increase in the branching reaction order *n* causes complete reduction in a reactive exothermic diffusion flow system as seen in the figures.

## 5. Conclusion

The criticality of a reactive generalized third-grade Couette fluid flow with Reynold's viscosity model is analyzed using the collocation weighted residual scheme. The thermal branch-chain criticality bifurcation, momentum, and temperature field solutions are analytically obtained. It is revealed from the investigation that

- The activation energy has no influence on the considered non-Newtonian fluid and temperature distribution in a Couette flow device
- It is obtained that, for engineering processes, the non-Newtonian reactive third-grade term enhances fluid viscosity by decreasing the flow rate
- From the aspect of industrial design, boundless heat production in the system due to the Frank-Kamenetskii term and initiation reaction rate can be controlled by exponential decrease in the fluid viscosity

The results will assist in combustion processes and in improving safety conditions of thermal explosion system. The solution algorithm is an interesting tool to study different parameter-dependent boundary-value nonlinear problems in engineering and science.

## Figures and Tables

**Table 1:** Comparison of results for the method of weighted residual collocation and numerical scheme.

*y*	*w*(*y*) (weighted residual results)	*w*(*y*) (numerical results)	Absolute error
−1.0	0	0	0
−0.8	0.2004037097	0.2004037157	6.0 × 10^−9^
−0.6	0.3901406612	0.3901406655	4.3 × 10^−8^
−0.4	0.5663565669	0.5663565721	5.2 × 10^−8^
−0.2	0.7257024719	0.7257024783	6.4 × 10^−8^
0.0	0.8639755022	0.8639755148	7.6 × 10^−8^
0.2	0.9754648475	0.9754648530	5.5 × 10^−8^
0.4	1.0518261984	1.0518262021	3.7 × 10^−8^
0.6	1.0819296871	1.0819296900	2.9 × 10^−8^
0.8	1.0610331196	1.0610331246	5.0 × 10^−9^
1.0	1.0000000000	1.0000000000	0

## Data Availability

No data were used to support this study.

## References

[1] Chinyoka T., Makinde O. D. (2013). Numerical investigation of entropy generation in unsteady MHD generalized couette flow with variable electrical conductivity. *The Scientific World Journal*.

[2] Salawu S. O., Oladejo N. K., Dada M. S. (2019). Analysis of unsteady viscous dissipative poiseuille fluid flow of two-step exothermic chemical reaction through a porous channel with convective cooling. *Ain Shams Engineering Journal*.

[3] Nayak M. K., Abdul Hakeem A. K., Ganga B., Ijaz Khan M., Waqas M., Makinde O. D. (2020). Entropy optimized MHD 3D nanomaterial of non-newtonian fluid: a combined approach to good absorber of solar energy and intensification of heat transport. *Computer Methods and Programs in Biomedicine*.

[4] Wang J., Ijaz Khan M., Khan W. A., Abbas S. Z., Imran Khan M. (2020). Transportation of heat generation/absorption and radiative heat flux in homogeneous-heterogeneous catalytic reactions of non-newtonian fluid (oldroyd-B model). *Computer Methods and Programs in Biomedicine*.

[5] Makinde O. D., Maserumule R. L. (2008). Thermal criticality and entropy analysis for a variable viscosity Couette flow. *Physica Scripta*.

[6] Farooq M., Rahim M. T., Islam S., Siddiqui A. M. (2013). Steady poiseuille flow and heat transfer of couple stress fluids between two parallel inclined plates with variable viscosity. *Journal of the Association of Arab Universities for Basic and Applied Sciences*.

[7] Hassan A. R., Gbadeyan J. A., Salawu S. O. (2018). The effects of thermal radiation on a reactive hydromagnetic internal heat generating fluid flow through parallel porous plates. *Springer Proceedings in Mathematics & Statistics*.

[8] Salawu S. O., Fatunmbi E. O., Ayanshola A. M. (2020). On the diffusion reaction of fourth-grade hydromagnetic fluid flow and thermal criticality in a plane couette medium. *Results in Engineering*.

[9] Yilbas B., Yürüsoy M., Pakdemirli M. (2004). Entropy analysis for non-newtonian fluid flow in annular pipe: constant viscosity case. *Entropy*.

[10] Yususoy M., Bayrakceken H., Kapucu M., Aksoy F. (2008). Entropy analysis for thirdgrade fluid flow vogel model viscosity in annular pipe. *International Journal of Non-Linear Mechanics*.

[11] Salawu S. O. (2018). Analysis of third-grade heat absorption hydromagnetic exothermic chemical reactive flow in a darcy-forchheimer porous medium with convective cooling. *WSEAS Transactions on Maths*.

[12] Adesanya S. O., Falade J. A., Jangili S., Anwar Bég O. (2017). Irreversibility analysis for reactive third-grade fluid flow and heat transfer with convective wall cooling. *Alexandria Engineering Journal*.

[13] Okoya S. S. (2019). Computational study of thermal influence in axial annular flow of a reactive third grade fluid with non-linear viscosity. *Alexandria Engineering Journal*.

[14] Khan M. I., Alzahrani F., Hobiny A. (2020). Simulation and modeling of second order velocity slip flow of micropolar ferrofluid with darcy-forchheimer porous medium. *Journal of Materials Research and Technology*.

[15] Salawu S. O., Disu A. B. (2020). Branch-chain criticality and thermal explosion of Oldroyd 6-constant fluid for a generalized couette reactive flow. *South African Journal of Chemical Engineering*.

[16] Okoya S. S. (2013). On criticality and disappearance of criticality for a branched-chain thermal reaction with distributed temperature. *Afrika Matematika*.

[17] Salawu S. O., Hassan A. R., Abolarinwa A., Oladejo N. K. (2019). Thermal stability and entropy generation of unsteady reactive hydromagnetic Powell-Eyring fluid with variable electrical and thermal conductivities. *Alexandria Engineering Journal*.

[18] Makinde O. D. (2009). Thermal criticality for a reactive gravity driven thin film flow of a third-grade fluid with adiabatic free surface down an inclined plane. *Applied Mathematics and Mechanics*.

[19] Frank-Kamenetskii D. A. (1969). *Diffusion and Heat Transfer in Chemical Kinetics*.

[20] Balakrishnan E., Swift A., Wake G. C. (1996). Critical values for some non-class a geometries in thermal ignition theory. *Mathematical and Computer Modelling*.

[21] Salawu S. O., Kareem R. A., Shonola S. A. (2019). Radiative thermal criticality and entropy generation of hydromagnetic reactive Powell-Eyring fluid in saturated porous media with variable conductivity. *Energy Reports*.

[22] Makinde O. D. (2006). Thermal ignition in a reactive viscous flow through a channel filled with a porous medium. *Journal of Heat Transfer*.

[23] Makinde O. D. (2004). Exothermic explosions in a slab: a case study of series summation technique. *International Communications in Heat and Mass Transfer*.

[24] Varatharajan B., Williams F. A. (2000). Ignition times in the theory of branched-chain thermal explosions. *Combust. Flame*.

[25] Truesdell C. A., Tuesdell C. A. (1965). On the foundations of mechanics of energies. *Continuum Mechanics II: The Rational Mechanics of Materials*.

[26] Fosdick R. L., Rajagopal K. R. (1980). Thermodynamics and stability of fluids of third grade. *in Proceedings of The Royal Society London A*.

[27] Salawu S. O., Ogunseye H. A., Olanrewaju A. M. (2018). Dynamical analysis of unsteady Poiseuille flow of two-step exothermic non-newtonian chemical reactive fluid with variable viscosity. *International Journal of Mechanical Engineering & Technology*.

[28] Okoya S. S. (2011). Disappearance of criticality for reactive third-grade fluid with Reynold’s model viscosity in a flat channel. *International Journal of Non-linear Mechanics*.

[29] Salawu S. O., Fatunmbi E. O. (2017). Inherent irreversibility of hydromagnetic third-grade reactive Poiseuille flow of a variable viscosity in porous media with convective cooling. *Journal of the Serbian Society for Computational Mechanics*.

[30] Boddington T., Feng C. G., Gray P. (1984). Thermal explosions, criticality, and the disappearance of criticality in systems with distributed temperatures II: an asymptotic analysis of criticality at the extremes of Biot number ((*Bi*⟶0), (*Bi*⟶*∞*)) for general reaction rate-laws. *in Proceedings of The Royal Society London A*.

[31] Salawu S. O., Dada M. S., Fenuga O. J. (2019). Thermal explosion and irreversibility of hydromagnetic reactive couple stress fluid with viscous dissipation and Navier slips. *Theoretical and Applied Mechanics Letters*.

[32] Salawu S. O., Abolarinwa A., Fenuga O. J. (2020). Transient analysis of radiative hydromagnetic Poiseuille fluid flow of two-step exothermic chemical reaction through a porous channel with convective cooling. *Journal of Computational and Applied Research in Mechanical Engineering*.

[33] Salawu S. O., Kareem R. A., Shamshuddin M. D., Khan S. U. (2020). Double exothermic reaction of viscous dissipative oldroyd 8-constant fluid and thermal ignition in a channel. *Chemical Physics Letters*.

[34] Salawu S. O., Fatunmbi E. O. (2020). Current density and criticality branch-chain for a reactive Poiseuille second-grade hydromagnetic flow with variable electrical conductivity. *International Journal of Thermofluids*.

